# Distinct characteristics of unique immunoregulatory canine non-conventional TCRαβ^pos^ CD4^neg^CD8α^neg^ double-negative T cell subpopulations

**DOI:** 10.3389/fimmu.2024.1439213

**Published:** 2024-08-09

**Authors:** Laura Karwig, Peter F. Moore, Gottfried Alber, Maria Eschke

**Affiliations:** ^1^ Institute of Immunology/Molecular Pathogenesis, Center for Biotechnology and Biomedicine, Faculty of Veterinary Medicine, Leipzig University, Leipzig, Germany; ^2^ Department of Veterinary Pathology, Microbiology and Immunology, School of Veterinary Medicine, University of California, Davis, Davis, CA, United States

**Keywords:** regulatory T cells, canine non-conventional Treg cells, TCRαβ^pos^ CD4^neg^ CD8^neg^ double-negative T cells, FoxP3, interleukin-10, peripheral blood

## Abstract

Conventional CD4^pos^ regulatory T (Treg) cells characterized by expression of the key transcription factor forkhead box P3 (FoxP3) are crucial to control immune responses, thereby maintaining homeostasis and self-tolerance. Within the substantial population of non-conventional T cell receptor (TCR)αβ^pos^ CD4^neg^CD8α^neg^ double-negative (dn) T cells of dogs, a novel FoxP3^pos^ Treg-like subset was described that, similar to conventional CD4^pos^ Treg cells, is characterized by high expression of CD25. Noteworthy, human and murine TCRαβ^pos^ regulatory dn T cells lack FoxP3. Immunosuppressive capacity of canine dn T cells was hypothesized based on expression of inhibitory molecules (interleukin (IL)-10, *cytotoxic T-lymphocyte associated protein 4, CTLA4)*. Here, to verify their regulatory function, the dnCD25^pos^ (enriched for FoxP3^pos^ Treg-like cells) and the dnCD25^neg^ fraction, were isolated by fluorescence-activated cell sorting from peripheral blood mononuclear cells (PBMC) of Beagle dogs and analyzed in an *in vitro* suppression assay in comparison to conventional CD4^pos^CD25^pos^ Treg cells (positive control) and CD4^pos^CD25^neg^ T cells (negative control). Canine dnCD25^pos^ T cells suppressed the Concanavalin A-driven proliferation of responder PBMC to a similar extent as conventional CD4^pos^CD25^pos^ Treg cells. Albeit to a lesser extent than FoxP3-enriched dn and CD4^pos^CD25^pos^ populations, even dnCD25^neg^ T cells reduced the proliferation of responder cells. This is remarkable, as dnCD25^neg^ T cells have a FoxP3^neg^ phenotype comparable to non-suppressive CD4^pos^CD25^neg^ T cells. Both, CD25^pos^ and CD25^neg^ dn T cells, can mediate suppression independent of cell-cell contact and do not require additional signals from CD4^pos^CD25^neg^ T cells to secrete inhibitory factors in contrast to CD4^pos^CD25^pos^ T cells. Neutralization of IL-10 completely abrogated the suppression by dnCD25^pos^ and CD4^pos^CD25^pos^ Treg cells in a Transwell™ system, while it only partially reduced suppression by dnCD25^neg^ T cells. Taken together, unique canine non-conventional dnCD25^pos^ FoxP3^pos^ Treg-like cells are potent suppressor cells *in vitro*. Moreover, inhibition of proliferation of responder T cells by the dnCD25^neg^ fraction indicates suppressive function of a subset of dn T cells even in the absence of FoxP3. The identification of unique immunoregulatory non-conventional dn T cell subpopulations of the dog *in vitro* is of high relevance, given the immunotherapeutic potential of manipulating regulatory T cell responses *in vivo.*

## 1 Introduction

The dog is not just a popular companion animal, but also an attractive model in immunological research. Autoimmune disorders (1), allergies (2, 3), and cancer (4, 5) regularly observed in dogs show similarities to these complex diseases in humans. Therefore, studies of pathogenesis mechanisms in dogs are not only valuable for the pet species itself but can also be useful in translational approaches to draw conclusions for human patients. In that regard, it is essential to intensify basic research on the canine immune system that is still poorly understood. Given the central role of T cells in immunity, a detailed knowledge of canine T cells can provide new insights into disease development and reveal new targets for therapies which could be eventually tested in this model species.

Across species, conventional T cells in the periphery express the T cell receptor (TCR)αβ and either CD8 or CD4 as a co-receptor. CD8 exists as CD8αα homodimer or as CD8αβ heterodimer, the latter being characteristic for conventional cytotoxic T cells (6). CD4 co-receptor expression is associated with T helper or regulatory T (Treg) cell function (7).

Besides conventional TCRαβ^+^ CD8 single-positive (sp) and CD4 sp T cells, a small population (1–3%) of non-conventional TCRαβ^+^ T cells that lack expression of both, CD4 and CD8, i.e. TCRαβ^pos^ CD4^neg^ CD8^neg^ double-negative (dn) T cells have been described in mice and humans (8–10). Of note, in dogs non-conventional TCRαβ^pos^ CD4^neg^CD8α^neg^ dn T cells constitute a large population of up to 15% of peripheral lymphocytes (11) raising the question regarding their function.

Conventional CD4^pos^ Treg cells defined by expression of the master transcription factor forkhead box P3 (FoxP3) are key players for maintenance of immune homeostasis and peripheral tolerance by preventing aberrant activation of the immune system (12–14). Treg cell dysfunction is associated with several common autoimmune disorders including multiple sclerosis, type 1 diabetes, rheumatoid arthritis, and systemic lupus erythematosus in humans (14). Furthermore, Treg cells have been implicated in allergic diseases and play a pivotal role in the maintenance of allograft tolerance (15, 16). Conventional CD4^pos^ FoxP3^pos^ Treg cells have also been described and functionally characterized in the dog (17, 18).

Recently, we identified a novel non-conventional FoxP3^pos^ Treg-like subset within the substantial population of canine CD4^neg^CD8α^neg^ dn T cells. Similar to conventional Treg cells, Treg-like dn T cells are characterized by high expression of the surface molecule CD25 (11). Potential immunosuppressive capacity of canine dn T cells was also indicated by their high stimulation-induced transcription of the co-inhibitory receptor *cytotoxic T-lymphocyte associated protein 4 (CTLA4)* and production of the inhibitory cytokine interleukin (IL)-10, as published by our group (19). The majority of IL-10-expressing dn T cells were CD25^pos^, corresponding to a T regulatory phenotype (19). Although a possible immunosuppressive function of FoxP3^pos^ dn T cells in the dog has been discussed, it has not yet been analyzed (17). Of note, allergen desensitization of dogs with adverse food reactions leads to an increase of canine dn T cells, supporting a potential regulatory role of canine dn T cells *in vivo* (20). However, dn T cells were not further characterized e.g. by expression of FoxP3 in this study.

Despite lacking FoxP3 expression (9, 10, 21–23), the small population of human and murine TCRαβ^pos^ dn T cells has been demonstrated to play an important role in down-regulating immune responses both *in vitro* and *in vivo* (24–29). In this context dn regulatory T cells of mice were able to prevent allograft rejection (9, 30), auto-immune diabetes (22, 31–34), or allergic asthma (35). Furthermore, a protective role of both, murine and human TCRαβ^pos^ dn T cells was shown in graft-versus-host disease (36–39). Based on recent data, it was speculated that dogs may have non-conventional FoxP3^pos^ and/or FoxP3^neg^ dn T cells with immunosuppressive function (19). To verify the hypothesis of a regulatory function of canine dn T cells in the present study, sorted CD25^pos^ and dnCD25^neg^ populations were assessed in *in vitro* suppression assays. We demonstrate that FoxP3-enriched dnCD25^pos^ T cells suppress proliferation of canine autologous responder peripheral blood mononuclear cells (PBMC) to a similar extent as conventional CD4^pos^ Treg cells. Furthermore, dnCD25^neg^ T cells but not conventional CD4^pos^CD25^neg^ T cells can suppress proliferation of PBMC responder populations. Non-conventional dnCD25^pos^ and dnCD25^neg^ T cells are able to mediate suppression via the secretion of soluble factors, with a central role of IL-10 in the former. In contrast to conventional CD4^pos^ Treg cells, non-conventional dn T cells do not require signals from co-cultured CD4^pos^CD25^neg^ T cells to exert their regulatory function in a Transwell™ system *in vitro*.

Taken together, our data prove existence of unique and potent suppressive subpopulations of non-conventional dn T cells in dogs that might play a crucial role in immune regulation *in vivo*.

## 2 Materials and methods

### 2.1 Dogs, blood sample collection

Peripheral blood samples were taken from healthy adult Beagle dogs via the lateral saphenous vein into heparinized vacutainer tubes (BD Vacutainer, 10ml, Li-Heparin 17 IU/ml, Becton Dickinson, Heidelberg, Germany). All laboratory dogs (5 male, 4 female, aged 2 - 8 years) belonged to the Faculty of Veterinary Medicine, Leipzig University, Germany, at the time of blood sampling. The number of dogs used for individual analyses is indicated in the figure legends. Dogs were vaccinated against common pathogens and prophylactically treated against endo- and ectoparasites on a regular basis. The study was authorized by the Animal Care and Usage Committee of the Saxony State Office, Leipzig, Germany (approval number: DD24.1–5131/444/30).

### 2.2 Isolation of canine peripheral blood mononuclear cells

Peripheral blood mononuclear cells (PBMC) were isolated from fresh whole blood by density gradient centrifugation within one hour after blood sampling. Briefly, blood was diluted with phosphate buffered saline (PBS) (ratio 1:1), layered above Pancoll^®^ Separating Solution (density 1.077 g/ml, PAN-Biotech, Aidenbach, Germany) and centrifuged (500g, 30 min at room temperature (RT) minimal acceleration and deceleration). Mononuclear cells from the interphase were collected and washed twice in PBS (500g, 15 min, RT). Cells were incubated for 5 min at RT in erythrocyte lysis buffer (150 mM NH_4_Cl, 8 mM KHCO_3_, 2 mM EDTA, pH 7.0). PBS with 3% fetal bovine serum (FBS, PAN-Biotech) was added to stop the reaction and remaining cells were washed again with PBS. The final cell number was determined using a Neubauer chamber (Laboroptik Lancing, UK), excluding dead cells by identification via trypan blue staining (Merck-Sigma, Darmstadt, Germany). PBMC were either used immediately for subsequent assays or were cryo-preserved in liquid nitrogen (in FBS + 10% DMSO) until further usage and used after thawing and washing in cell culture medium (IMDM with L-Glutamine, 25 mM HEPES and 3.024 g/L NaHCO_3_ (PAN-Biotech) supplemented with 100 U/ml penicillin, 100 μg/ml streptomycin (both purchased from PAA Laboratories, Cölbe, Germany), and 10% FBS).

A part of the isolated PBMC from each individual dog was labeled with Cell Proliferation Dye eFluor™ 450 (Thermo Fisher Scientific, Carlsbad, USA) according to the manufacturer’s protocol. These proliferation dye-labeled PBMC were used as responder (Resp) cells in *in vitro* suppression assays (see below).

### 2.3 Sort of effector T cell subpopulations

Remaining PBMC (see 2.2) from each individual dog were used to isolate effector T cell populations (Teff) by fluorescence-activated cell sorting (FACS). All incubation steps described below were performed for 15–20 min on ice in the dark and separated by washing steps with PBS, 3% FBS (500g, 3 min, 4°C). For discrimination between viable and dead cells, a fixable viability dye (eFluor 506, Thermo Fisher Scientific) was applied following the manufacturer’s protocol. Detailed information on the primary antibodies used for flow cytometric staining is summarized in **Table 1**. To avoid binding by Fc receptors, cells were incubated with heat-inactivated serum derived from dog and rat (15% each in PBS). Then, cells were incubated with anti-canine TCRαβ (clone CA15.8G7) hybridoma supernatant. A PerCP/Cy5.5-conjugated goat-anti-mouse IgG secondary antibody (Biolegend, San Diego, USA) was used for detection. In a next step, cells were incubated with a mixture of the remaining cell surface antibodies, listed in **Table 1**. Stained cells were passed over a 30 µm filter (Sysmex Germany GmbH, Norderstedt, Germany) and immediately sorted using a BD FACSAria™ III Cell Sorter equipped with BD FACS Diva ™ software version 6.1.3 (Becton Dickinson).

**Table 1:** Antibodies used for flow cytometric analysis.

*Specificity*	*Clone*	*Isotype*	*Species* *reactivity*	*Source*	*Fluorochrome*
TCRαβ	CA15.8G7	mouse IgG1	dog	Leukocyte Antigen Biology Laboratory, Davis, USA	None (hybridoma supernatant)
Anti-mouse IgG	Poly4053	goat polyclonal IgG	mouse	Biolegend, San Diego, USA	Peridinin chlorophyll protein-Cyanine5.5 (PerCP/Cy5.5)
CD4	YKIX302.9	rat IgG2a	dog	Thermo Fisher Scientific, Carlsbad, USA Bio-Rad, Munich, Germany	Allophycocyanin (APC)
CD8α	YCATE55.9	rat IgG1	dog	Bio-Rad, Munich, Germany	Fluorescein (FITC), Alexa Fluor 700
CD25	P4A10	mouse IgG1	dog	Thermo Fisher Scientific, Carlsbad, USA	Phycoerythrin (PE)
FoxP3	FJK-16s	rat IgG2a	mouse/rat, cross-reactivity with dog (40–43)	Thermo Fisher Scientific, Carlsbad, USA	Alexa Fluor 488

### 2.4 Analysis of sorted T cell subpopulations

The portion of FoxP3^pos^ cells within each sorted Teff population was determined using the FoxP3/Transcription Factor Staining Buffer Set (Thermo Fisher Scientific) according to the manufacturer’s protocol. After fixation and permeabilization, a mixture of heat-inactivated dog, rat and mouse normal serum (15% each in permeabilization buffer) was added to block binding by Fc receptors and cells were stained with the anti-canine FoxP3 antibody in permeabilzation buffer (Thermo Fisher Scientific) for 30 minutes at RT in the dark. Data were acquired with a BD LSR Fortessa™ Cell Analyzer equipped with FACS Diva™ software version 6.1.3 (Becton Dickinson) and assessed using the FlowJo 10™ software (Treestar Inc.).

### 2.5 *In vitro* suppression assay

Resp cells and sorted Teff cells were resuspended in cell culture medium and final cell numbers were determined using a Neubauer chamber, taking into account cell loss through FACS-sorting and staining procedures. 5x10^4^ Resp cells were seeded into 96-well round bottom cell culture plates (Techno Plastic Products AG, Trasadingen, Switzerland) and stimulated with 0.5–1.0 µg/ml Concanavalin A (ConA) (Roth, Karlsruhe, Germany) to reach a proliferation rate of >25% that enables assessment of suppressive effects by Teff cells. For analysis of their suppressive capacity, the different autologous sorted Teff cells were added to proliferation dye-labelled Resp cells at a ratio of 1:1. Cells were incubated for 72 h at 37°C, 5% CO_2_ in a total volume of 200 µl culture medium. Resp cells alone incubated with medium or stimulated with ConA in parallel served as controls.

To allow for analysis of different T responder cell (Tresp) populations, the mixed cells were harvested after three days of culture, and re-stained for TCRαβ, CD4, and CD8α. Briefly, after washing, LIVE/DEAD™ Fixable Orange (602) Viability Kit (Thermo Fisher Scientific) was used following the manufacturer’s protocol. Staining for surface antibodies, data acquisition and analysis were performed as described above.

The suppressive effect of purified Teff cells on the proliferation of the different responder T cell populations was normalized to ConA-stimulated Tresp only and calculated as follows (18):


$$\text{\% suppression }=\frac{\text{\% of proliferated “Tresp only ConA” }-\text{ \% of proliferated “Tresp ConA in presence of Teff”}}{\text{\% of proliferated “Tresp only ConA”}}\times \;100.$$

### 2.6 *In vitro* Transwell™ suppression assay

Transwell™ experiments were performed in 96-well plates (Corning Incorporated Costar, Tewksbury, USA) to determine if cell-cell contact is required to mediate suppressive effects. As described for co-culture suppression assays, proliferation dye-labeled Resp cells were seeded at a density of 5x10^4^ cells in the bottom chamber of the Transwell™ system and stimulated with 0.4 µg/ml ConA. Cells assayed for their regulatory capacity (Teff) were cultured at an equal ratio in the top chamber, separated from Tresp via a 0.4 µm pore polycarbonate membrane. Teff cells were either cultured alone in the Transwell™ insert, or co-cultured with CD4^pos^CD25^neg^ cells at a ratio of 1:1 in order to compare suppressive effects with and without contact to CD4^pos^CD25^neg^ cells. To analyze whether IL-10 is involved in cell-cell contact independent suppression, a neutralizing anti-canine IL-10 antibody (clone 138128, R&D, Biotechne, Wiesbaden, Germany) was used at 10 µg/ml. A nonblocking mouse IgG1 antibody (clone 11711, R&D, Biotechne, Wiesbaden, Germany) was added as control to rule out unspecific effects of the neutralizing antibody. Similar to the standard co-culture suppression assay, cells were incubated for 72 h at 37°C, 5% CO_2_. Analysis of suppression was performed as described above.

### 2.7 Statistical analysis

The Graph Pad Prism10 software (GraphPad Software Inc., San Diego, USA) was used for statistical analysis and for graphical presentation of the data. The Shapiro-Wilk test was applied to test for normal distribution. Normally distributed data are presented with means, and were analyzed via One-way ANOVA with Bonferroni *post hoc* test. Non-parametric data are depicted with medians, and were compared by Kruskal-Wallis test with Dunn’s post test. (* p< 0.05; ** p< 0.01, *** p< 0.001). Significance of antibody-dependent effects (anti-IL-10 or isotype control) on suppression was calculated by direct comparison with the corresponding control without antibody addition using the Mann-Whitney test (two-tailed), (* p< 0.05).

## 3 Results

### 3.1 Canine non-conventional TCRαβ^pos^ CD4^neg^CD8α^neg^ double-negative (dn)CD25^pos^ T cells are potent suppressors *in vitro*, and even dnCD25^neg^ T cells act suppressive

**Figure 1:**
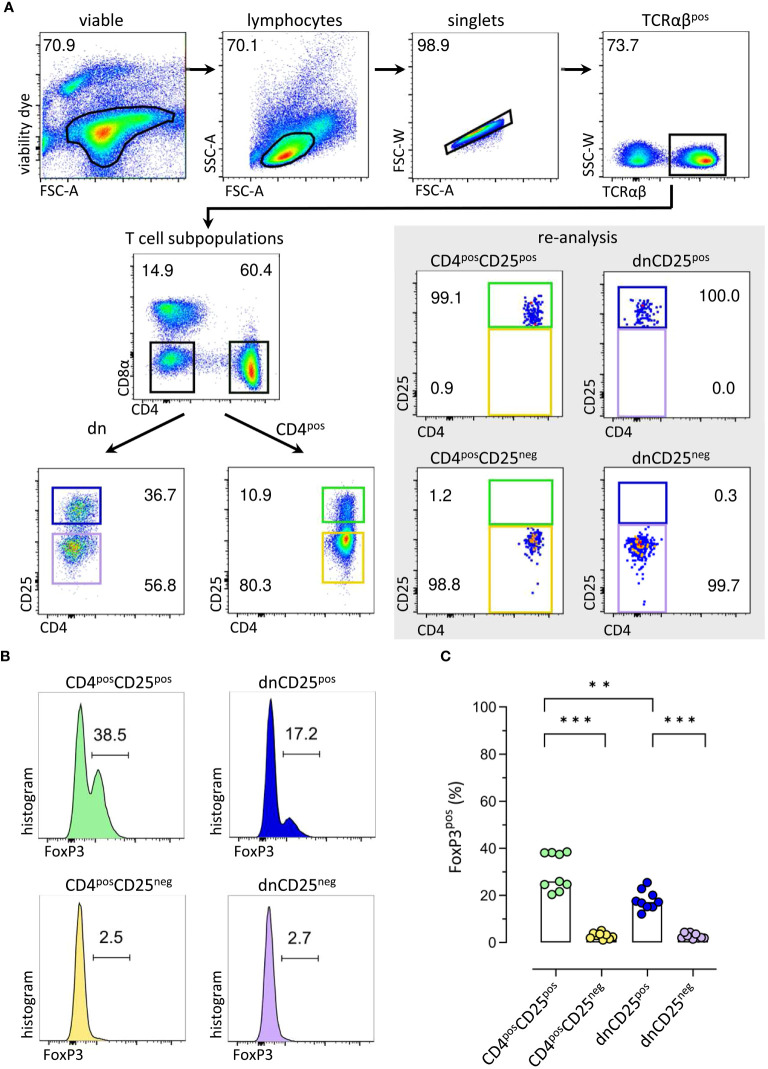
Sorting of canine T effector (Teff) cells for comparative analysis of conventional and non-conventional populations in an *in vitro* suppression assay results in enrichment of FoxP3^pos^ cells in CD4^pos^CD25^pos^ and CD4^neg^CD8α^neg^ double-negative (dn)CD25^pos^ TCRαβ^pos^ T cell fractions. **(A)** The strategy for isolation of different Teff cell populations from canine peripheral blood mononuclear cells (PBMC) by fluorescence-activated cell sorting is shown. After gating on single viable TCRαβ^pos^ lymphocytes, conventional CD4^pos^ single-positive and non-conventional CD4^neg^CD8α^neg^ double-negative (dn) T cells were further divided according to CD25 expression to obtain four Teff populations: CD4^pos^CD25^pos^, CD4^pos^CD25^neg^, dnCD25^pos^, dnCD25^neg^. High purity (>98%) of the isolated populations was confirmed by re-analysis. Representative data of one dog are depicted. Gated populations’ percentages are presented as numbers in the plots. **(B, C)** FoxP3 expression in sorted Teff populations demonstrates that FoxP3^pos^ cells are enriched in the CD4^pos^CD25^pos^ and dnCD25^pos^ Teff populations. **(B)** Representative flow cytometric plots show the expression of FoxP3 (%) in the sorted Teff populations indicated. Gating was performed using CD8α^pos^ T cells as internal control. **(C)** Quantification of the frequencies of FoxP3^pos^ cells in the different sorted Teff populations. Each dot represents one individual dog analysed in independent experiments. Bars represent medians. Statistical analysis was done by Kruskal-Wallis test with Dunn’s post-test (** *p*< 0.01, *** *p*<0.001).

**Figure 2:**
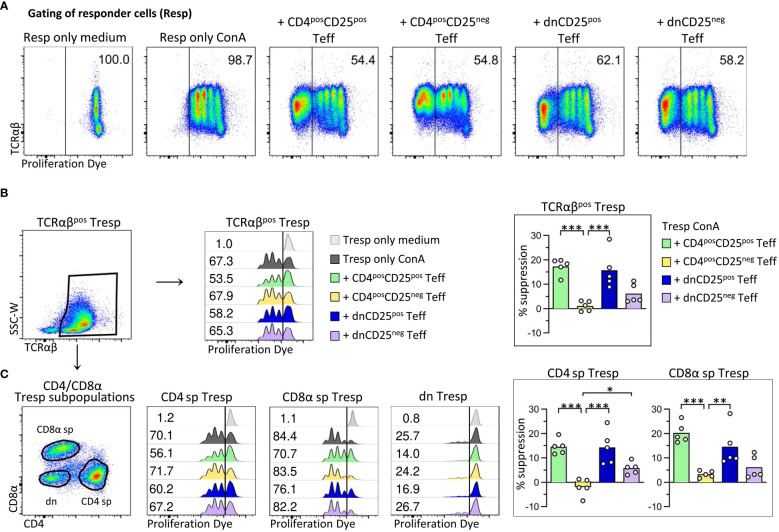
Canine non-conventional FoxP3-enriched CD4^neg^CD8a^neg^ double-negative (dn)CD25^pos^ T cells, and, to a lesser extent, dnCD25^neg^ T cells suppress the proliferation of autologous responder PBMC *in vitro*. Proliferation dye-labelled responder PBMC (Resp) were co-cultured with indicated sorted effector T cell (Teff) populations (CD4^pos^CD25^pos^, CD4^pos^CD25^neg^, dnCD25^pos^, dnCD25^neg^) at an effector: responder ratio of 1:1 and stimulated with Concanavalin A (ConA). Responder cells cultured alone under medium or ConA conditions served as controls. After three days of culture, the proliferation of different responder T cell populations was evaluated by flow cytometry and the suppressive effect of Teff cells was calculated. **(A)** From viable single lymphocytes gated as shown in **Figure 1A**, proliferation dye-negative Teff cells were excluded and responder cells (Resp) selected for analysis of proliferation. The gate was set based on ConA-stimulated responder cells cultured alone (Resp only ConA). Representative data of one dog are shown with the frequency of responder cells under all conditions. **(B, C)** Whole PBMC as responder cells enabled analysis of suppressive effects of Teff cells on the proliferation of different responder cell populations, i.e. **(B)** all TCRαβ^pos^ responder cells (Tresp), and **(C)** TCRαβ^pos^ CD4^pos^ (CD4 sp) and CD8α^pos^ (CD8α sp) single-positive as well as CD4^neg^CD8α^neg^ double-negative (dn) Tresp subpopulations. (detailed gating strategy in **Supplementary Figure 3** for all co-culture conditions) **(B, C)** Flow cytometric histograms of one representative dog show the proliferation frequencies of indicated Tresp populations, co-cultured with indicated Teff cell population (see color code). The gate was set according to the unstimulated negative control (Resp only medium). Note that the low proliferation rate of dn Tresp upon ConA stimulation did not allow reliable quantification of suppressive effects on these. But suppression was quantified for TCRαβ^pos^ Tresp **(B)** as well as for CD4 sp and CD8α sp Tresp **(C)**. Summary bar graphs **(B, C)** show the percent suppression of proliferation of the indicated Tresp population, mediated by the different Teff cell population (see color code). The percentage of suppression was normalized to ConA-stimulated Tresp only and calculated as follows: (% of proliferated “Tresp only ConA” − % of proliferated “Tresp ConA co-cultured with indicated Teff”)/(% of proliferated “Tresp only ConA”) × 100. Each dot represents one individual dog analysed in independent experiments. Data are presented with the mean and statistical analysis was performed using One-way ANOVA with Bonferroni’s post-test (* *p*< 0.05; ** *p*< 0.01, *** *p*< 0.001). (**Supplementary Figure 4** shows suppressive effects mediated by different Teff cell populations on TCRαβ^pos^ Tresp, compared to all viable lymphocytes as responder cells (Resp).).

To assess their suppressive function *in vitro*, TCRαβ^pos^ dnCD25^pos^ and dnCD25^neg^ subpopulations were isolated by fluorescence-activated cell sorting from peripheral blood of healthy Beagle dogs with high purity (>98.0%) (**Figure 1A**). Conventional CD4^pos^CD25^pos^ Treg and CD4^pos^CD25^neg^ cells were sorted as positive and negative control, respectively (17, 18) (**Figure 1A**). CD25^pos^ and CD25^neg^ T cell subpopulations were defined by use of a control staining including all antibodies except for CD25 which was replaced by its isotype control (**Supplementary Figure 1**). Consistent with our previous results, non-conventional TCRαβ^pos^ dn T cells contained an approximately three times higher proportion of CD25^pos^ cells than their conventional CD4^pos^ counterparts [**Figure 1A** (11)]. As expected, FoxP3^pos^ cells were enriched in both, the conventional CD4^pos^CD25^pos^ fraction [median 30% FoxP3^pos^ (17)] and the dnCD25^pos^ fraction [median 18% FoxP3^pos^ (11)], as shown by post-sort re-analysis (**Figures 1B, C**). In contrast, a comparable, mainly FoxP3^neg^ phenotype was observed in sorted CD4^pos^CD25^neg^ and dnCD25^neg^ populations (median<3% FoxP3^pos^). Interestingly, post-sort re-analysis revealed a significantly lower expression of TCRαβ in both non-conventional dn T cell subpopulations, compared to their conventional CD4^pos^ counterparts (**Supplementary Figure 2**). The sorted T effector (Teff) cell populations’ regulatory capacities were assessed in an *in vitro* suppression assay. Autologous proliferation dye-labeled responder (Resp) PBMC were stimulated with ConA for three days in the presence or absence of sorted Teff cells (**Figure 2A**). Suppressive effects on all TCRαβ^pos^ T (Tresp) cells (**Figure 2B**) and individual Tresp cell subpopulations, i.e. CD4 single-positive (sp), CD8α sp and dn Tresp cells (**Figure 2C**) were analyzed (see **Supplementary Figure 3** for detailed gating strategy). The proliferative response was similar when responder cells were gated for all viable lymphocytes or the dominating TCRαβ^pos^ population (**Supplementary Figure 4**). Noteworthy, while ConA induced strong proliferation of CD4 sp and CD8α sp Tresp cells, the proliferation rate of non-conventional dn Tresp cells was very low (**Figure 2C**) and did not allow for reliable quantification of suppressive effects of Teff cells in this subpopulation.

As expected (17, 18), canine conventional CD4^pos^CD25^pos^ Treg cells suppressed proliferation of autologous CD4 sp Tresp cells (mean suppression 15%) (**Figures 2B, C**). Furthermore, proliferation of CD8α^pos^ Tresp cells was suppressed by 20% in the presence of CD4^pos^CD25^pos^ Teff cells, expanding the knowledge on target cells of canine conventional CD4^pos^ Treg cells.

Importantly, canine non-conventional TCRαβ^pos^ dnCD25^pos^ Teff cells suppressed the ConA-driven proliferation of Tresp cells to a similar extent as conventional CD4^pos^CD25^pos^ Treg cells (mean suppression 15%) (**Figures 2B, C**). DnCD25^pos^-mediated suppression of proliferation was observed for TCRαβ^pos^ CD4^pos^ Tresp and TCRαβ^pos^ CD8α^pos^ Tresp cells (**Figure 2C**).

Remarkably, despite having a comparable FoxP3^neg^ phenotype as non-suppressive CD4^pos^CD25^neg^ T cells (**Figures 1B, C**), dnCD25^neg^ Teff cells also reduced proliferation of Tresp cells (mean suppression 6%), albeit to a lesser extent than the FoxP3-enriched dnCD25^pos^ and CD4^pos^CD25^pos^ populations (**Figures 2B, C**).

Taken together, these findings clearly indicate that, besides conventional CD4^pos^CD25^pos^ Treg cells, the canine immune system contains circulating non-conventional immunoregulatory TCRαβ^pos^ dn T cell subpopulations, i.e. FoxP3-enriched dnCD25^pos^ T cells, and, less suppressive, dnCD25^neg^ T cells.

### 3.2 Canine non-conventional TCRαβ^pos^ CD4^neg^CD8α^neg^ double-negative (dn) T cells secrete inhibitory molecules in a cell-cell contact independent manner

**Figure 3:**
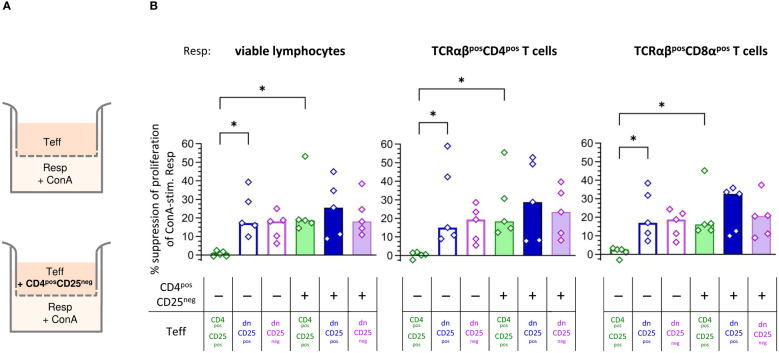
Canine non-conventional CD4^neg^CD8a^neg^ double-negative (dn)CD25^pos^ and dnCD25^neg^ T cells suppress responder cells independent of cell-cell contact *in vitro*. **(A)** Experimental setup of the suppression assay in the Transwell™ system (schematic): indicated effector T (Teff) cell populations, assayed for their suppressive capacity, were cultured in the top well of a 96-well Transwell™ plate and separated from ConA-stimulated proliferation dye-labelled responder cells (Resp) in the bottom chamber by a 0.4 µm semi-permeable membrane that only allows soluble factors to pass through. Teff cells in the top chamber were cultured as separate Teff, or co-cultured with the same number of CD4^pos^CD25^neg^ cells (indicated ‘+’) to test for the requirement of cell-cell contact to induce production of suppressive soluble factors in the different Teff cell populations (CD4^pos^CD25^pos^, dnCD25^pos^, dnCD25^neg^). After three days of incubation, Resp cells were harvested from the bottom chamber and analysed by flow cytometry. **(B)** The suppression of proliferation of Resp cells, mediated by CD4^pos^CD25^pos^, dnCD25^pos^ and dnCD25^neg^ Teff cells (see color code) with/without contact to CD4^pos^CD25^neg^ T cells was calculated by normalization to the respective negative control (i.e. stimulated Resp, that were cultured under separation from non-suppressive CD4^pos^CD25^neg^ Teff cells by the Transwell™ membrane). The suppressive effects on viable lymphocytes, TCRαβ^pos^ CD4^pos^ and TCRαβ^pos^ CD8α populations are shown. Each dot represents cells from one individual dog analysed in five independent experiments, bars represent medians. Statistical analysis was performed using Kruskal-Wallis test with Dunn’s post test. (* *p*< 0.05).

Next, we were interested in the mechanisms by which TCRαβ^pos^ dn T cells suppress Resp cells’ proliferation. We first asked whether the observed suppression depended on cell-cell contact and/or secretion of soluble factors. Transwell™ suppression assays, in which Teff cells are separated from Resp cells by a semi-permeable membrane that prevents cell-cell contact, while diffusion of soluble factors is maintained (**Figure 3A**, upper panel), are a suitable method to address this question (44–46).

Human, murine and porcine conventional CD4^pos^CD25^pos^ Treg cells do not suppress proliferation of Resp cells when physically separated (45–52). Similarly, when cultured alone in the Transwell™ insert, suppressive capacity of canine CD4^pos^CD25^pos^ Teff cells was completely abolished (**Figure 3B**). In striking contrast, both dnCD25^pos^ and dnCD25^neg^ Teff populations mediated suppression independent of cell-cell contact in the Transwell™ system (**Figure 3B**). Secretion of suppressive molecules by murine conventional CD4^pos^CD25^pos^ Treg cells is dependent on co-culture with other cells, such as CD4^pos^CD25^neg^ cells (44). To investigate the impact of signals from other cells on the suppressive capacity of the Teff cells by secretion of inhibitory factors, they were cultured in the upper chamber in presence of non-suppressive CD4^pos^CD25^neg^ T cells (**Figure 3A**, lower panel). Indeed, the addition of CD4^pos^CD25^neg^ T cells (+ columns in **Figure 3B**) significantly restored suppression by CD4^pos^CD25^pos^ Teff cells in the Transwell™ system (**Figure 3B**). Thus, secretion of suppressive soluble factors by canine conventional CD4^pos^CD25^pos^ Treg cells depends on activation signals from other cells in close proximity. In contrast, contact to CD4^pos^CD25^neg^ cells in the Transwell™ system did not significantly increase suppressive effects of dnCD25^pos^ and dnCD25^neg^ Teff cells showing intrinsic capacity to secrete inhibitory molecules (**Figure 3B**).

Taken together, canine dnCD25^pos^ and dnCD25^neg^ can mediate suppression independent of cell-cell contact via secretion of inhibitory factors. Induction of suppression mediated by both non-conventional dn Teff cell populations clearly differs from that of conventional CD4^pos^CD25^pos^ Tregs, as, in contrast to the latter, they do not require interaction with co-cultured CD4^pos^CD25^neg^ T cells to secrete suppressive soluble factors in the Transwell™ system.

### 3.3 IL-10 is an important suppressive mediator of canine non-conventional CD4^neg^CD8α^neg^ double-negative (dn)CD25^pos^ and conventional CD4^pos^CD25^pos^ T cells in the Transwell™ system

**Figure 4:**
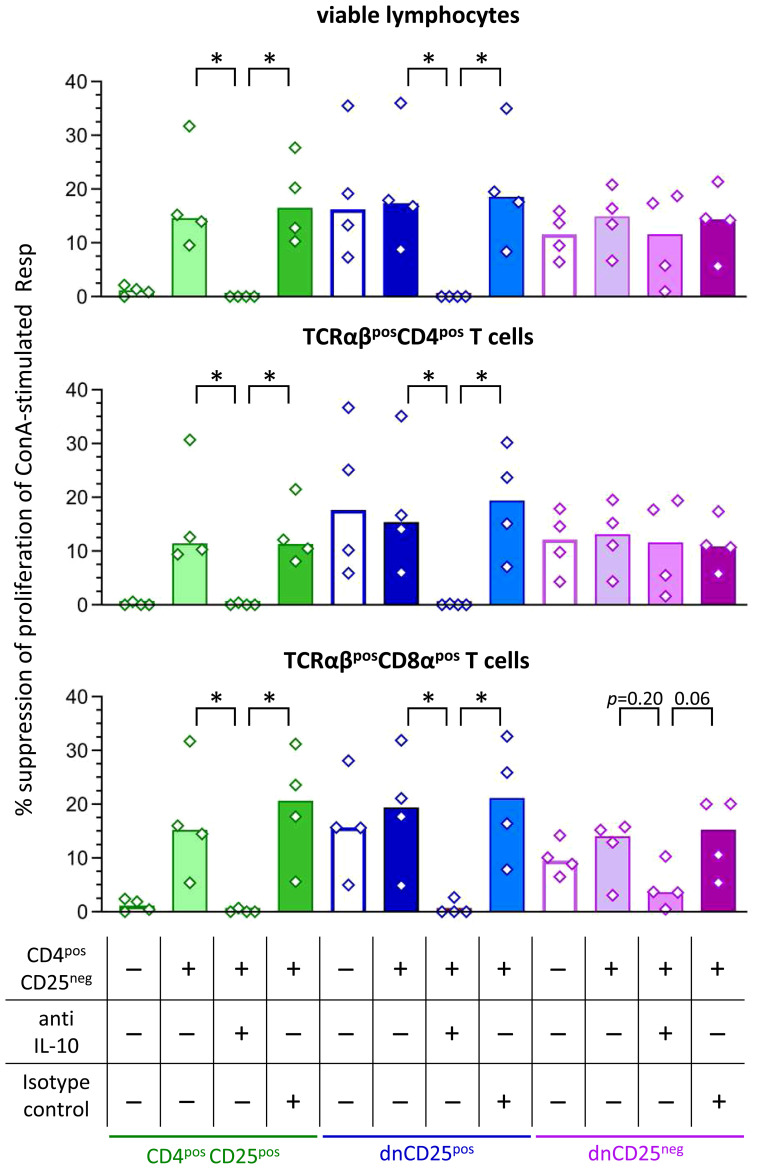
Secretion of IL-10 by canine dnCD25^pos^ and CD4^pos^CD25^pos^ Teff cells is necessary to mediate suppression in the Transwell™ system. Suppression assays were performed in the Transwell™ system as described in **Figure 3A**. Proliferation dye-labeled ConA-stimulated responder (Resp) cells were cultured in the bottom well, separated by a semipermeable membrane from indicated Teff cell populations in the top well. Where indicated, CD4^pos^CD25^neg^ cells were added at a ratio of 1:1 to Teff cells (top well). The IL-10-dependency of suppression was assessed by addition of a neutralizing anti-IL-10 antibody in comparison to a non-blocking isotype control antibody (see table). Resp cells were harvested after 72 h and analysed by flow cytometry. Summary bar graphs show the median suppression of proliferation of viable lymphocytes, TCRαβ^pos^ CD4^pos^ and TCRαβ^pos^ CD8α^pos^ Tresp mediated by the different Teff cell population under each condition (see table). The percentage of suppression was normalized to the negative control (ConA stimulated Resp + CD4^pos^CD25^neg^ Teff) of each condition (+/- CD4^pos^CD25^neg^, +/- anti IL-10 antibody, +/- isotype control). In case addition of neutralizing IL-10 antibody led to negative values for suppression (i.e. ablation of suppression), values are depicted as 0% suppression. Notably, neutralization of IL-10 completely abrogates the suppression by dnCD25^pos^ and CD4^pos^CD25^pos^ Teff cells in the Transwell™ system, while it only partially reduces the suppression by dnCD25^neg^ Teff cells. The results of three independent experiments are shown. Each dot represents one individual dog, bars represent medians. Significance of antibody-dependent effects (anti-IL-10) on suppression was calculated by direct comparison with the corresponding controls (i.e. control without antibody addition and isotype control) using the Mann-Whitney test (two-tailed). (* *p* < 0.05).

Non-conventional TCRαβ^pos^ dn T cells of the dog are potent IL-10 producers *in vitro*, similar to their CD4^pos^ counterparts (19). To determine whether IL-10 plays a role for cell-contact independent suppression of proliferation mediated by the non-conventional dn T cell subpopulations as well as conventional CD4^pos^CD25^pos^ T cells, a neutralizing anti-IL-10 antibody was added to the upper chamber of the Transwell™ system. A non-neutralizing isotype control antibody was used for control. As CD4^pos^CD25^pos^ T cells need contact to CD4^pos^CD25^neg^ T cells to secrete inhibitory factors (**Figure 3B**), we decided to use the experimental set-up including these together with the Teff cells (compare lower panel of **Figure 3A**). Of note, the addition of the neutralizing anti-IL-10 antibody completely abrogated suppression by dnCD25^pos^ Teff cells in the Transwell™ system, while their suppressive activity was unaltered in the presence of the isotype control antibody (**Figure 4**). Similar results were obtained for CD4^pos^CD25^pos^ Teff cells. The addition of the neutralizing anti-IL-10 antibody specifically abolished suppression mediated across the semi-permeable membrane (**Figure 4**). Taken together, these data clearly show that IL-10 is necessary to mediate suppression by canine dnCD25^pos^ and CD4^pos^CD25^pos^ Teff cells in the Transwell™ system. Although a tendency towards reduced suppressive effects of dnCD25^neg^ Teff cells on TCRαβ^pos^ CD8α^pos^ Tresp cells was observed, neutralization of IL-10 did not significantly reduce suppression by dnCD25^neg^ Teff cells (**Figure 4**), indicating that other soluble suppressive mediators may be secreted by this non-conventional subpopulation.

## 4 Discussion

The role of conventional CD4^pos^FoxP3^pos^ Treg cells is well recognized as guardians of the immune system, to maintain tolerance, and to prevent autoimmunity (53–55). Furthermore, human and murine non-conventional TCRαβ^pos^ CD4^neg^CD8α^neg^ dn T cells have been shown to be suppressive and play an important role in down-regulation of immune responses both *in vitro* and *in vivo* (9, 21, 24–29).

The relatively rare TCRαβ^pos^ dn T cells of men and mice are universally FoxP3^neg^ (9, 10, 21–23), but dogs’ PBMC contain a substantial population of TCRαβ^pos^ dn T cells including a subset expressing FoxP3 (11). Immunosuppressive capacity of canine dn T cells was hypothesized based on high stimulation-induced upregulation of inhibitory molecules, including IL-10 (19).

Using an *in vitro* suppression assay here, we proved the existence of unique immunoregulatory TCRαβ^pos^ dn T cell subpopulations of the dog, which clearly differ from their murine and human counterparts (**Table 2**). We demonstrated that canine FoxP3-enriched dnCD25^pos^ T cells suppress the ConA-driven proliferation of responder cells to a similar extent as conventional CD4^pos^ Treg cells *in vitro*. Albeit to a lesser extent, dnCD25^neg^ T cells are also suppressive despite a FoxP3^neg^ phenotype comparable to non-suppressive CD4^pos^CD25^neg^ T cells. Irrespective of this similarity to murine and human FoxP3^neg^ dn T cells, canine dnCD25^neg^ T cells were found to be distinct in terms of activation requirements and suppressive mechanisms. **Table 2** summarizes functional similarities and differences between human, murine and canine non-conventional TCRαβ^pos^ dn T cells, as well as conventional CD4^pos^ Treg cells.

**Table 2:** Unique features of immunosuppressive double-negative (dn) T cells and conventional regulatory T cells of the dog *in vitro* in comparison to their murine and human counterparts.

	Non-conventional double-negative (dn) T cells (TCRαβ^pos^ CD4^neg^CD8a^neg^ dn)		Conventional Treg cells (TCRαβ^pos^ CD4^pos^CD25^pos^)	
Species	Mouse, human	Dog	Dog	Mouse, human
CD25 phenotype	dn**CD25^neg^**	dn**CD25^pos^** and dn**CD25^neg^**	CD4^pos^ **CD25^pos^**	CD4^pos^ **CD25^pos^**
Expression of **FoxP3**	no (9, 10)	dnCD25^pos^: yes (11)	yes (17, 18)	yes (56–58)
**Mechanism of suppression:**				
Pre-activation necessary for suppression in co-culture	yes (9, 21)	no	no	no (50, 51)
Suppression by secretion of soluble molecules in the Transwell™ system: - without additional signals from CD4^pos^CD25^neg^ cells - with additional signals from CD4^pos^CD25^neg^ cells	no (9, 21, 59–61) not analyzed	yes yes	no yes	no (46–52) mouse: yes (44) human: not analyzed
IL-10-mediated suppression *in vitro*	no (21, 61)	dnCD25^pos^: yes	yes	no (46, 50–52)

While freshly isolated human and murine dn T cells are unable to suppress responder cells under resting conditions *in vitro* (9, 21), canine dnCD25^pos^ and dnCD25^neg^ T cells do not need pre-activation before co-culture to exert their suppressive function. Using the Transwell™ system, we could show that both, canine non-conventional dnCD25^pos^ and dnCD25^neg^ T cells are able to mediate suppression cell-cell contact independently via secretion of inhibitory molecules. In striking contrast, despite a diverse spectrum of inhibitory mechanisms (12, 13, 49, 53, 54, 62), suppression by human and murine dn T cells requires cell–cell contact and is not solely mediated by soluble factors (9, 21, 59–61). The secretion of soluble immunosuppressive factors, such as IL-10, TGF-β (63, 64), and IL-35 (65) is an important mechanism of suppression by conventional CD4^pos^ Treg cells *in vivo* (53, 54). Noteworthy, we could show that, similar to murine Treg cells (44), canine conventional CD4^pos^ Treg cells need signals from other cells (CD4^pos^CD25^neg^) to secrete suppressive molecules *in vitro*. In contrast, non-conventional dnCD25^pos^ and dnCD25^neg^ T cells of the dog seem to have intrinsic capacity to secrete inhibitory factors as indicated by our Transwell™ experiments. Neutralization of IL-10 in the Transwell™ system completely abrogated suppression mediated by canine dnCD25^pos^ T cells, suggesting that IL-10 is a highly relevant cytokine for cell-cell contact independent suppression of proliferation by this subpopulation *in vitro*. In contrast, IL-10 does not play a role for suppression by murine (9, 26) and human non-conventional dn T cells *in vitro*. Although human dn T cells secrete some IL-10 (10, 21, 66, 67), addition of a neutralizing anti-IL-10 antibody was not able to abrogate their suppressive activity in co-culture experiments (21). Similarly, neutralization of IL-10 did not alter suppressive effects of murine and human conventional CD4^pos^ Treg cells *in vitro* (46, 50–52). Noteworthy, we could show that canine conventional CD4^pos^CD25^pos^ Treg cells differ from their murine and human counterparts and use IL-10 secretion as a potent suppression mechanism *in vitro*. In contrast, the suppressive effects of dnCD25^neg^ T cells did not rely on IL-10 secretion. Whether other inhibitory soluble factors or synergistic effects between different cytokines play a role in canine cells needs further investigation. Synergism has been shown for TGF-β and IL-10 in mice (68, 69).

In conclusion, our data demonstrate that canine CD25^pos^ and CD25^neg^ TCRαβ^pos^ CD4^neg^CD8α^neg^ dn T cells are potent suppressor cells *in vitro* with unique features compared to dn T cells of mice and humans as well as conventional CD4^pos^ Treg cells (summarized in **Table 2**). This provides the basis for future *in vivo* studies addressing the role of canine non-conventional immunoregulatory dn T cell subpopulations in health and disease, which may ultimately inform new immunotherapeutic strategies.

## Data Availability

The original contributions presented in the study are included in the article/**Supplementary Material**. Further inquiries can be directed to the corresponding author.
