# Supplementary material for: Distinct characteristics of unique immunoregulatory canine non-conventional TCRαβpos CD4negCD8αneg double-negative T cell subpopulations
Source: Front Immunol. 2024 Aug 9;15:1439213. doi: 10.3389/fimmu.2024.1439213 (PMC11341405; doi:10.3389/fimmu.2024.1439213)
Supplement: Supplementary file 1 [file Presentation_1.pptx]

## Slide 1
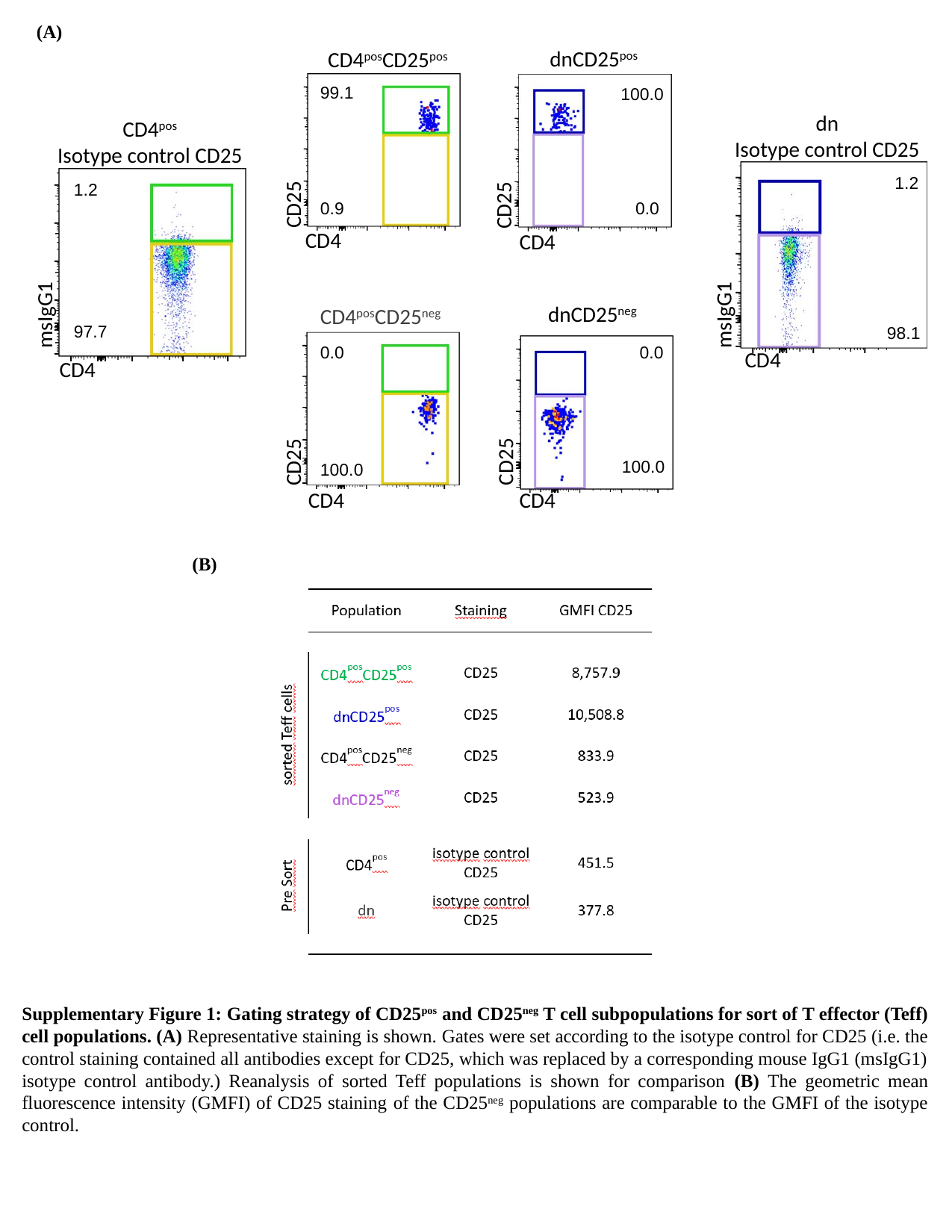

(A)
dnCD25pos
CD4posCD25pos
99.1
100.0
dn
Isotype control CD25
CD4pos
Isotype control CD25
CD25
CD25
1.2
1.2
0.9
0.0
CD4
CD4
msIgG1
msIgG1
dnCD25neg
CD4posCD25neg
97.7
98.1
0.0
0.0
CD4
CD4
CD25
CD25
100.0
100.0
CD4
CD4
(B)
Supplementary Figure 1: Gating strategy of CD25pos and CD25neg T cell subpopulations for sort of T effector (Teff) cell populations. (A) Representative staining is shown. Gates were set according to the isotype control for CD25 (i.e. the control staining contained all antibodies except for CD25, which was replaced by a corresponding mouse IgG1 (msIgG1) isotype control antibody.) Reanalysis of sorted Teff populations is shown for comparison (B) The geometric mean fluorescence intensity (GMFI) of CD25 staining of the CD25neg populations are comparable to the GMFI of the isotype control.

## Slide 2
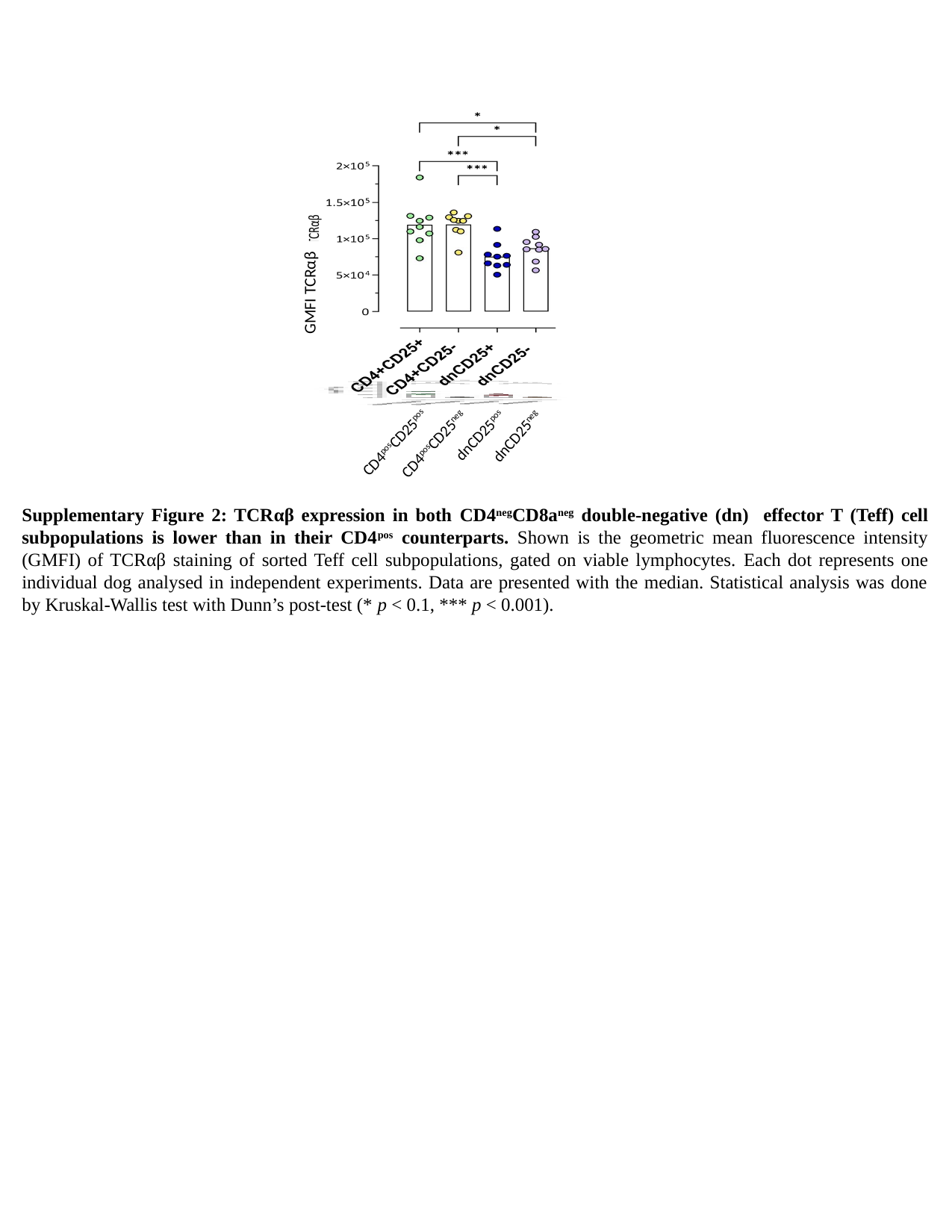

GMFI TCRαβ
CD4posCD25pos
dnCD25pos
CD4posCD25neg
dnCD25neg
Supplementary Figure 2: TCRαβ expression in both CD4negCD8aneg double-negative (dn) effector T (Teff) cell subpopulations is lower than in their CD4pos counterparts. Shown is the geometric mean fluorescence intensity (GMFI) of TCRαβ staining of sorted Teff cell subpopulations, gated on viable lymphocytes. Each dot represents one individual dog analysed in independent experiments. Data are presented with the median. Statistical analysis was done by Kruskal-Wallis test with Dunn’s post-test (* p < 0.1, *** p < 0.001).

## Slide 3
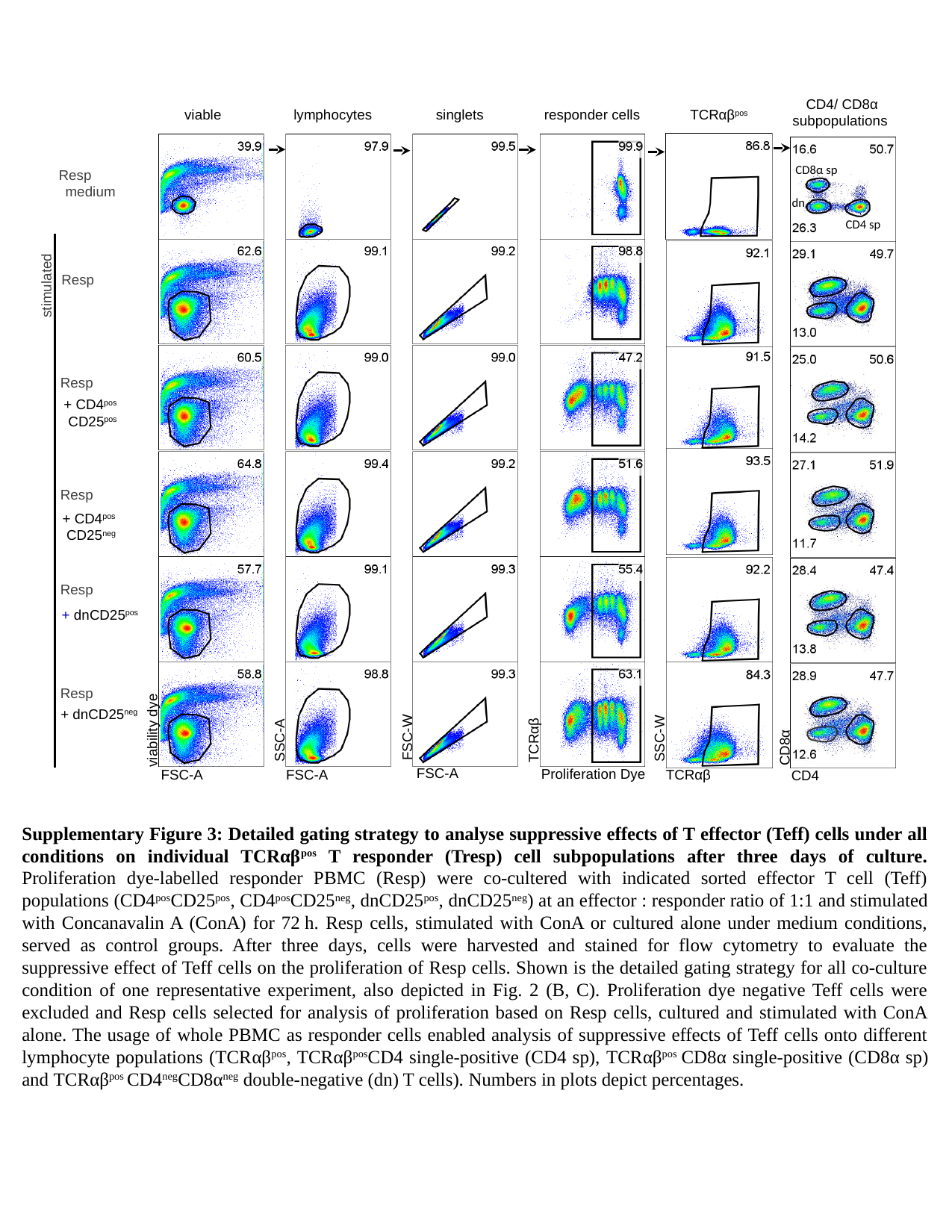

CD4/ CD8α
subpopulations
viable
lymphocytes
singlets
responder cells
TCRαβpos
CD8α sp
Resp
medium
dn
CD4 sp
Resp
stimulated
Resp
+ CD4pos
 CD25pos
Resp
+ CD4pos
 CD25neg
Resp
+ dnCD25pos
Resp
FSC-W
SSC-A
SSC-W
TCRαβ
CD8α
+ dnCD25neg
viability dye
FSC-A
Proliferation Dye
TCRαβ
FSC-A
FSC-A
CD4
Supplementary Figure 3: Detailed gating strategy to analyse suppressive effects of T effector (Teff) cells under all conditions on individual TCRαβpos T responder (Tresp) cell subpopulations after three days of culture. Proliferation dye-labelled responder PBMC (Resp) were co-cultered with indicated sorted effector T cell (Teff) populations (CD4posCD25pos, CD4posCD25neg, dnCD25pos, dnCD25neg) at an effector : responder ratio of 1:1 and stimulated with Concanavalin A (ConA) for 72 h. Resp cells, stimulated with ConA or cultured alone under medium conditions, served as control groups. After three days, cells were harvested and stained for flow cytometry to evaluate the suppressive effect of Teff cells on the proliferation of Resp cells. Shown is the detailed gating strategy for all co-culture condition of one representative experiment, also depicted in Fig. 2 (B, C). Proliferation dye negative Teff cells were excluded and Resp cells selected for analysis of proliferation based on Resp cells, cultured and stimulated with ConA alone. The usage of whole PBMC as responder cells enabled analysis of suppressive effects of Teff cells onto different lymphocyte populations (TCRαβpos, TCRαβposCD4 single-positive (CD4 sp), TCRαβpos CD8α single-positive (CD8α sp) and TCRαβpos CD4negCD8αneg double-negative (dn) T cells). Numbers in plots depict percentages.

## Slide 4
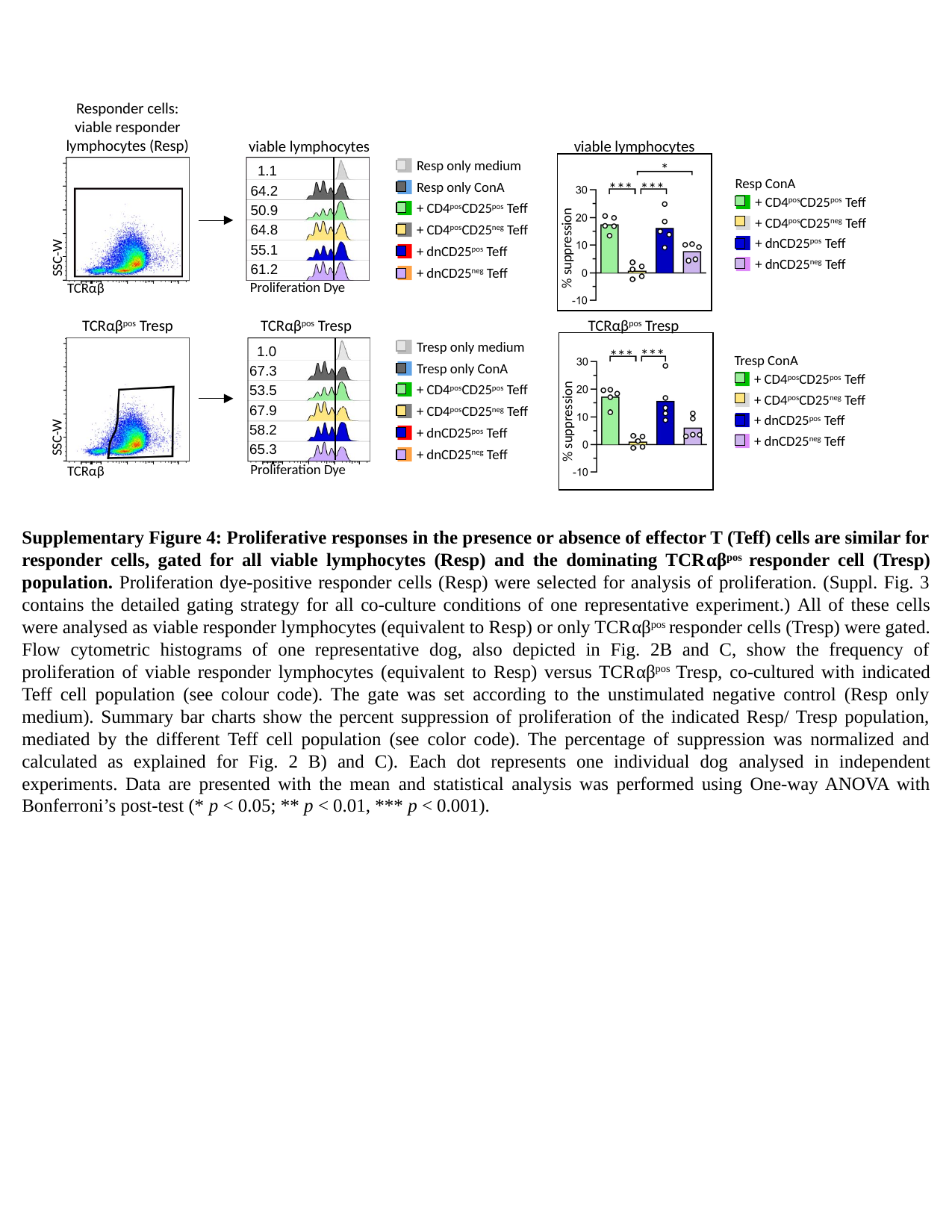

Responder cells:
viable responder lymphocytes (Resp)
viable lymphocytes
viable lymphocytes
■ Resp only medium
■ Resp only ConA
■ + CD4posCD25pos Teff
■ + CD4posCD25neg Teff
■ + dnCD25pos Teff
■ + dnCD25neg Teff
*
***
***
% suppression
1.1
64.2
50.9
64.8
55.1
61.2
Proliferation Dye
Resp ConA
■ + CD4posCD25pos Teff
■ + CD4posCD25neg Teff
■ + dnCD25pos Teff
■ + dnCD25neg Teff
SSC-W
TCRαβ
TCRαβpos Tresp
TCRαβpos Tresp
TCRαβpos Tresp
■ Tresp only medium
■ Tresp only ConA
■ + CD4posCD25pos Teff
■ + CD4posCD25neg Teff
■ + dnCD25pos Teff
■ + dnCD25neg Teff
1.0
67.3
53.5
67.9
58.2
65.3
***
***
SSC-W
TCRαβ
Tresp ConA
■ + CD4posCD25pos Teff
■ + CD4posCD25neg Teff
■ + dnCD25pos Teff
■ + dnCD25neg Teff
% suppression
Proliferation Dye
Supplementary Figure 4: Proliferative responses in the presence or absence of effector T (Teff) cells are similar for responder cells, gated for all viable lymphocytes (Resp) and the dominating TCRαβpos responder cell (Tresp) population. Proliferation dye-positive responder cells (Resp) were selected for analysis of proliferation. (Suppl. Fig. 3 contains the detailed gating strategy for all co-culture conditions of one representative experiment.) All of these cells were analysed as viable responder lymphocytes (equivalent to Resp) or only TCRαβpos responder cells (Tresp) were gated. Flow cytometric histograms of one representative dog, also depicted in Fig. 2B and C, show the frequency of proliferation of viable responder lymphocytes (equivalent to Resp) versus TCRαβpos Tresp, co-cultured with indicated Teff cell population (see colour code). The gate was set according to the unstimulated negative control (Resp only medium). Summary bar charts show the percent suppression of proliferation of the indicated Resp/ Tresp population, mediated by the different Teff cell population (see color code). The percentage of suppression was normalized and calculated as explained for Fig. 2 B) and C). Each dot represents one individual dog analysed in independent experiments. Data are presented with the mean and statistical analysis was performed using One-way ANOVA with Bonferroni’s post-test (* p < 0.05; ** p < 0.01, *** p < 0.001).
